# Risk of new endocrinological deficits after intraoperative MRI-guided additional resection in endoscopic non-functioning pituitary adenoma surgery

**DOI:** 10.1007/s11102-026-01727-0

**Published:** 2026-07-11

**Authors:** Stefanos Voglis, Meltem Gönel, Maria Triantafyllidou, Victor Staartjes, Gianluca Mosca, Jacopo Bellomo, Benjamin Beyersdorf, Kevin Akeret, Bas van Niftrik, Michael Hugelshofer, Bettina Winzeler, Zoran Erlic, Luca Regli, Carlo Serra

**Affiliations:** 1 Department of Neurosurgery, Clinical Neuroscience Center, University Hospital, University of Zurich, Zurich, Switzerland; 2 Department of Endocrinology, University Hospital and University of Zurich, Zurich, Switzerland; 3 Present Address: Department of Neurosurgery, University Hospital Frankfurt, Frankfurt, Germany

**Keywords:** Intraoperative MRI, Pituitary adenoma, Extent of resection, Endocrinological deficits, Outcome

## Abstract

**Objective:**

Intraoperative magnetic resonance imaging (ioMRI) has been increasingly used in transsphenoidal surgery for pituitary adenomas to improve the rate of gross total resection (GTR). However, its influence on postoperative pituitary function—particularly the risk of new endocrinological deficits (EDs) due to an additional resection—has not been investigated in detail. This study aimed to evaluate the endocrinological outcomes of ioMRI-guided TSS in patients with nonfunctioning pituitary adenomas (NFPAs), with a focus on the risk of new EDs following ioMRI-guided additional resection.

**Methods:**

We performed a retrospective cohort analysis of prospectively collected data from 312 patients who underwent endoscopic transsphenoidal surgery with the “chopsticks” technique between July 2013 and July 2022. Of these, 155 patients met the inclusion criteria: histologically confirmed NFPA phenotype and 3-Tesla ioMRI usage. All patients had at least 3 months of endocrinological and MRI follow-up. Hormonal outcomes were reviewed by a dedicated endocrinologist using clinical and biochemical assessments.

**Results:**

Among the 155 included patients (median age 60 years; 59% male), the ioMRI GTR rate was 35%; this increased to 65% (*n* = 98) on 3‑month postoperative MRI as a result of ioMRI‑guided additional resection. Overall, additional resection following ioMRI was performed in 46% (*n* = 71) of cases. New EDs of at least one pituitary axis were observed in 23% (*n* = 35) of patients at discharge, 25% (*n* = 38) at first follow-up (6 weeks postoperatively), and 18% (*n* = 28) at final follow-up in total. Any recovery of preoperative EDs occurred in 9.7%, 31% and 45% respectively. Uni- and multivariate logistic regression analysis showed that neither GTR, tumor volume, residual tumor volume nor additional resection following ioMRI were associated with increased risk of new deficits.

**Conclusions:**

IoMRI-guided additional resection during TSS for NFPAs can be performed safely without increasing the risk of new EDs. These findings support the utility of ioMRI in maximizing the extent of resection while preserving pituitary function.

**Supplementary Information:**

The online version contains supplementary material available at https://doi.org/10.1007/s11102-026-01727-0.

## Introduction

Pituitary neuroendocrine tumors, commonly referred to as pituitary adenomas, are among the most frequent intracranial neoplasms, comprising approximately 10% of all intracranial tumors, with an estimated prevalence of up to 16.7% in the population [1]. While generally benign and slow growing, these tumors can result in significant morbidity through either hormonal hypersecretion or local mass effect on surrounding structures. Nonfunctioning pituitary adenomas (NFPAs) are the most prevalent subtype and are often diagnosed later due to an absence of obvious endocrine symptoms. Diagnosis is typically established either in the context of a mass effect caused by a macroadenoma or, increasingly often, when imaging is performed for unrelated reasons.

Transsphenoidal endoscopic surgery (TSS) is the primary treatment for patients with symptomatic or progressively enlarging NFPAs [2]. The primary surgical objective is to achieve a gross total resection (GTR) while preserving normal pituitary function. Intraoperative magnetic resonance imaging (ioMRI) is routinely employed in many centers to optimize extent of resection while maintaining safety. This strategy has been shown to significantly increase the GTR rate [3] indicating that approximately 20% of patients initially judged to have a subtotal resection (STR) could be converted to a GTR due to additional resection based on ioMRI findings.

Despite the recognized benefits of ioMRI in achieving an increased rate of GTR [3–6], there is no consensus on its influence on postoperative pituitary function, particularly regarding the risk of inducing new pituitary hormone deficiencies through additional resection after ioMRI. The primary concern is that an additional resection prompted by ioMRI may lead to more aggressive manipulation within the surgical cavity, thereby increasing the risk of new endocrinological impairments. This closely parallels the paradigm in glioma surgery, where the specific association of ioMRI-guided additional resection on new postoperative neurological deficits has been investigated in recent years [7, 8].

This study aims to evaluate the endocrinological outcomes of ioMRI-guided TSS for NFPAs and determine if additional resection following ioMRI increases the risk of new endocrinological deficits (EDs).

## Methods

### Study design and patient selection

All consecutive patients who underwent TSS for NFPA performed at the Department of Neurosurgery, University Hospital Zurich between July 2013 and July 2022 were included in the study cohort. Cases where no 3-Tesla ioMRI was used were excluded (mainly due to logistic or patient-specific reasons, see Supplementary Fig. 1 for the study cohort inclusion flow-chart).

## Surgical technique and intraoperative 3-Tesla MRI

All procedures were performed endoscopically using the “chopsticks” technique, a mononostril, bimanual endoscopic approach, as previously described [9]. A detailed description of the surgical workflow according to the Pituitary society expert Delphi consensus can be found in the Supplementary Methods [10].

Intraoperative assessment of extent of resection was performed using a high-field 3-Tesla ioMRI (3T Skyra VD13, Siemens). ioMRI sequences were assessed together with experienced, board-certified neuroradiologists for tumor remnants and the decision to continue the resection in cases with residual tumor was based on individual patient characteristics (e.g. age, planned resection goal, co-morbidities).

## MRI and volumetric analyses

NFPA characteristics were determined using pre- (within 3 months preoperatively) intra- and postoperative (3 month postoperatively) T1-weighted MRI images after gadolinium contrast (volumetric analysis, classifications).

The Zurich Pituitary Radio (Maximum horizontal adenoma diameter / Minimum intercarotid distance) and score [11, 12] was routinely calculated as a thoroughly validated indicator for surgical outcomes. Volumetric measurements were done by an experienced neurosurgeon and intra- as well as for postoperative images were assessed by a board-certified neuroradiologist. All adenoma volumes were measured using Smartbrush (Brainlab AG, Munich, Germany). GTR was assessed based on the 3-months postoperative MRI.

## Endocrinological evaluation

Endocrinologic evaluation was routinely performed in an outpatient setting before and after surgery and during regular follow-up. The first postoperative control was scheduled 6 weeks after discharge and additional follow-up visits were based on individual assessments. Median first follow-up timepoint in the cohort was 6 weeks (interquartile range, IQR 4.9–8.9 weeks) and for last follow-up 4.1 years (IQR 2–6 years). For the endocrinological assessment prior to and after surgery, we retrospectively assessed clinical reports from the in-hospital and outpatient endocrinological visits, including clinical evaluation and laboratory data, according to standard clinical practice.

A new ED was defined as a newly manifest hormonal deficiency compared to baseline that required hormone replacement therapy at the respective follow-up timepoint, distinguishing early transient postoperative disturbances at discharge from permanent deficiencies at mid- and long-term follow-up. Recovery of a preoperative deficiency was defined as the absence of need for hormonal replacement therapy at the latest postoperative visit, without fulfilling the criteria for deficiency as described below.

For the purpose of this study, preoperative corticotrope deficiency was defined either by the need for initiation of hydrocortisone substitution by the treating endocrinologist prior to surgery, or by the presence of clinical signs and symptoms associated with a morning cortisol level < 300 nmol/L, or by a morning cortisol level < 200 nmol/L even in the absence of documented clinical signs or symptoms, after exclusion of potential influencing factors (e.g. exogenous glucocorticoid treatment, hormonal contraception). The initiation of perioperative prophylactic hydrocortisone treatment alone was not considered as deficiency. Postoperatively, the persistent need for hydrocortisone substitution at each endocrinological visit or the need for initiation of treatment defined corticotrope insufficiency, based on the same criteria as before surgery.

For thyreotrope insufficiency, we considered the need for pre- or postoperative substitution according to the treating endocrinologist’s decision, or the presence of clinical signs and symptoms of hypothyroidism associated with an fT4 value below the normal range, or an fT4 value < 10 pmol/L if no clinical data were available, with a low or inadequately not elevated TSH level. Patients under treatment for primary hypothyroidism prior to surgery were excluded from the evaluation of thyreotrope deficiency.

Gonadotrope insufficiency was defined by the clinical presence of oligo- or amenorrhea in premenopausal women, by inadequately low gonadotropin levels in postmenopausal women, or by morning testosterone levels below the reference range with inappropriately low (within or below the normal range) gonadotropin levels in men. Gonadotrope insufficiency was not assessed in patients under hormonal contraception prior to or after surgery.

For somatotrope insufficiency, we considered patients with substitution treatment according to the treating endocrinologist or a IGF-1 below reference level. No dynamic testing was performed for somatotrope function.

AVP deficiency (central diabetes insipidus) was defined by postoperative new onset of polyuria and polydipsia or by elevated sodium and serum osmolality. It was defined as transient if no hormonal replacement therapy (desmopressin) was required after discharge, or as persistent AVP deficiency if treatment remained necessary.

If data fulfilling the above criteria were not available from the clinical records, the respective variables were considered missing and excluded from further analyses.

## Additional resection criteria and propensity score matching analysis

The intraoperative decision to perform an additional surgical resection following ioMRI was individualized and based on a combination of baseline clinical and radiographic characteristics. This decision was primarily guided by the preoperatively intended extent of resection (e.g., gross total resection vs. intended subtotal resection), patient age, and the anatomical location of the tumor remnant on ioMRI. Notably, residual tumor within the cavernous sinus was usually left unresected in NFPA cases.

To control for potential selection bias inherent to these non-randomized surgical decisions, a 1:1 nearest-neighbor propensity score matching analysis was conducted. Propensity scores were calculated using a logistic regression model that included the following covariates: preoperatively intended GTR, patient age, and the presence of residual tumor in the cavernous sinus. Covariate balance between the matched “additional resection” and “no additional resection” sub-cohorts was verified via standardized mean differences (Supplementary Fig. 2). Univariate and multivariate logistic regression analyses were then performed within the matched cohort subset to evaluate the robustness of our primary outcome findings. Propensity score matching was performed in R utilizing the *MatchIt* package.

### Data acquisition and statistical analysis

Clinicopathologic, imaging, and periprocedural characteristics and follow-up data of each patient were extracted from the electronic clinical information in accordance with the international Delphi consensus core outcome set for pituitary surgery [13], wherever the respective recordings were available. All data processing and statistical analyses were performed using R Studio (version 1.4, R Studio Inc.) with open-source libraries. P-values < 0.05 were considered statistically significant. Statistical tests are indicated in the main text and figure captions. Due to the low number of target events (new endocrinological deficits), standard multivariate logistic regression risks overfitting. Therefore, we performed Firth’s penalized likelihood logistic regression using the *logistf* package in R to obtain stable estimates and control for confounding. Variables with missing values were handled using complete-case analysis; given the low proportion of missingness per variable (the large majority below 5%, see Supplementary Table 1) and no systematic differences between cases with missing variables and complete ones, missing data points were considered random and therefore no imputation was performed. Raw data and R scripts for the figures and analyses are available from the corresponding author upon reasonable request.

## Ethical considerations

Data collection and study analysis were approved by the local Ethics Review Board (“Kantonale Ethikkommission Zürich”, identifiers: 2015 − 0142 and 2017-00093) and registered at clinicaltrials.gov (NCT01628406), individual patient consent was waived, and patient data were treated in accordance with local ethical regulations and the Declaration of Helsinki.

## Results

### Patient cohort characteristics

From a total of 312 patients who underwent TSS for NFPA between July 2013 and July 2022, 116 were excluded due to non–NFPA phenotype. Of the remaining 196 patients 41 were excluded due to the absence of ioMRI. The final study cohort consisted of 155 patients who met the inclusion criteria (Supplementary Fig. 1). The median age was 60 years (SD ± 13), and 92 patients (59%) were male. Most surgeries (91%) were first-time transsphenoidal procedures, and 16 patients (11%) presented with preoperative apoplexy (see Table 1 for detailed patient characteristics).

**Table 1:** Baseline patient and tumor characteristics

Characteristic	
Sex	
Female	63 (41%)
Male	92 (59%)
Age (mean ± SD)	60 (13)
First TSS	
No	13 (8.9%)
Yes	133 (91%)
Unknown	9
Apoplexy	
No	128 (89%)
Yes	16 (11%)
Unknown	11
Phenotype	
NFPA	155 (100%)
Zurich Pituitary Score	
I	13 (8.4%)
II	93 (60%)
III	39 (25%)
IV	9 (5.8%)
Unknown	1
Knosp score	
0	14 (9.1%)
1	47 (31%)
2	48 (31%)
3 A	26 (17%)
3B	8 (5.2%)
4	11 (7.1%)
Unknown	1
Tumor volume preop [ml]	6.8 (6.8)
unknown	3
Additional resection after ioMRI	
No	84 (54%)
Yes	71 (46%)
Intended extent of resection	
decompression	3 (1.9%)
GTR	120 (78%)
STR	31 (20%)
unknown	1
Extent of resection [%] at 3 months	0.96 (0.11)
unknown	5
Extent of resection at 3 months	
GTR	98 (65%)
STR	52 (35%)
unknown	5

*TSS transsphenoidal surgery; NFPA non-functioning pituitary adenoma; GTR gross total resection; STR subtotal resection*

### Tumor characteristics

Tumor invasiveness was assessed using the Knosp grading system [14], with most tumors classified as Knosp grade 1 or 2 (62%, Table 1). Based on the Zurich Pituitary Score [12] which predicts the likelihood of transsphenoidal GTR, 60% of patients were categorized as Zurich Pituitary Score II (Table 1) and the mean preoperative tumor volume was 6.8 ml.

### Surgical characteristics, extent of resection and ioMRI assessment

**Fig. 1:**
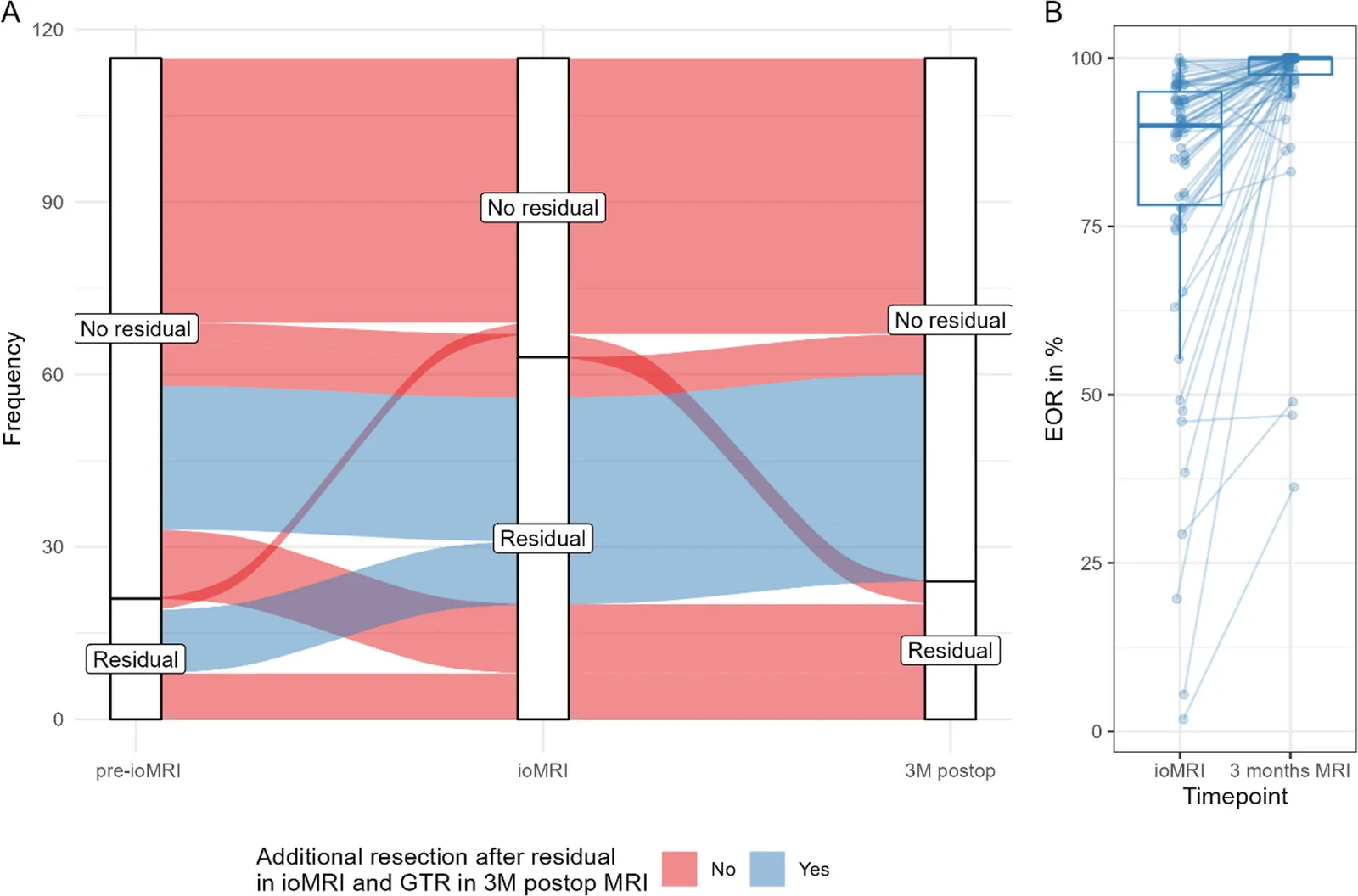
Gross total resection outcomes of additional resection following residual tumor detection in ioMRI

The overall GTR rate was 35% in ioMRI, which increased to 65% upon additional resection in the 3 month postoperative MRI. Overall, an additional resection after ioMR was performed in 46% (*n* = 71) of cases. Of the patients for whom the surgeon intended GTR, 54.8% (*n* = 63) showed evidence of residual tumor in ioMRI. Of those patients, 73.5% achieved GTR following ioMRI-guided additional resection (Fig. 1). Assessment of the tumor remnants on ioMRI showed that most remnants were located suprasellar and intrasellar, followed by those extending into the cavernous sinus (see Supplementary Fig. 3 for the anatomical distributions and volumes of residual tumors on ioMRI). Volumetric analysis of residual tumors showed an increase in mean extent of resection from 82% to 96% for cases with additional resection, illustrating the improved extent of resection upon ioMRI-guided additional resection (Fig. 1B). At 3-month follow-up MRI, 65% of the cases in which GTR was intended by the surgeon (which were 78% of the entire cohort), showed GTR of the adenoma (Table 1).

Intraoperative cerebrospinal fluid (CSF) leak occurred in 27%, while postoperative CSF fistulas were observed in 3.3% of cases (Table 2). There were no intraoperative vascular injuries, worsening of visual acuity deficits or postoperative infections recorded. Furthermore, during follow-up there were no re-admissions and no re-operations or other interventions (e.g. radiotherapy or systemic therapy) due to recurrent tumor.

**Table 2:** Complications and endocrinological outcomes

Characteristic	
CSF leak intraop	41/154 (27%)
CSF fistula postop	5/153 (3.3%)
Medical complication	
none	152/155 (98%)
SIADH	3/155 (1.9%)
New endocrinological deficit	
at discharge	35/154 (23%)
at first FU	38/154 (25%)
at last FU	28/154 (18%)
Any endocrinological recovery:	
at discharge	10/103 (9.7%)
at first FU	32/104 (31%)
at first FU	47/105 (45%)

*CSF cerebrospinal fluid; SIADH syndrome of inappropriate antidiuretic hormone release; FU follow-up*

### Endocrinological outcome

**Fig. 2:**
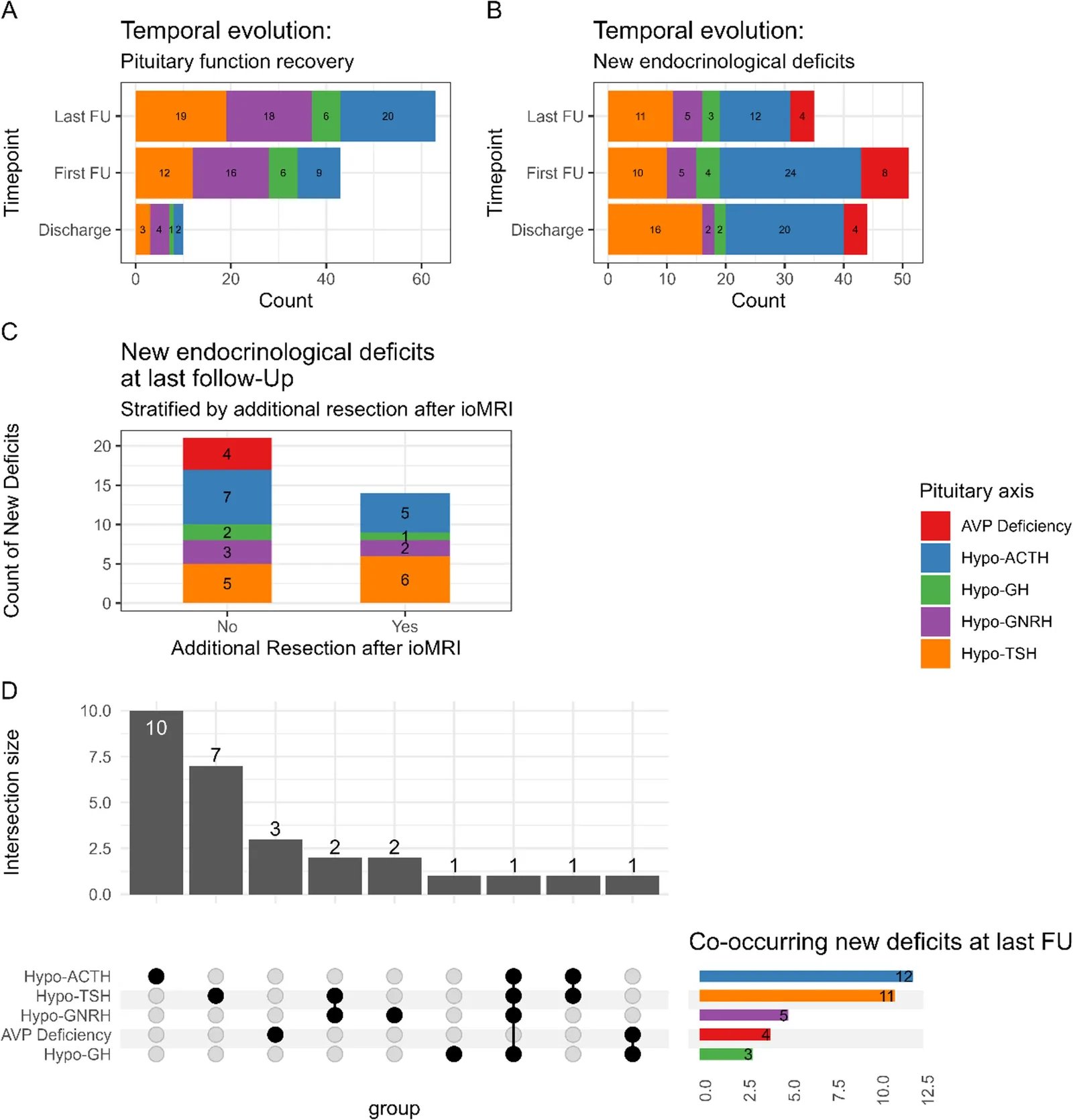
Endocrinological outcomes on the respective hypothalamic-pituitary-adrenal axis

Recovery of any preoperative EDs (in total, 110 patients had any ED preoperatively) increased at the later follow-up timepoints from 9.7% (*n* = 10) at discharge, 31% (*n* = 32) at first FU to 45% (*n* = 47) at last FU (Table 2; with a median last follow-up time of 4.1 years, IQR 2–6 years). The most pronounced recoveries were observed in the corticotropic, thyreotropic, and gonadotropic axes (Fig. 2A). There was no significant difference between endocrinological recoveries of patients which underwent additional resection after ioMRI compared to those who did not (Supplementary Table 2).

On the other hand, new EDs of at least one axis were observed in 23% of patients at discharge, in 25% at the first postoperative follow-up, and in 18% at the final follow-up (Table 2). Again, there was no significant difference between new EDs of patients which underwent additional resection after ioMRI compared to those who did not (Supplementary Table 3).

Of the patients with new EDs, those deficits at discharge affected most commonly the corticotropic axis (*n* = 20, 45.5% of new EDs), followed by the thyrotropic axis (*n* = 16, 36.4%), with lower rates observed in the somatotropic (*n* = 2, 4.5%) and gonadotropic (*n* = 2, 4.5%) axes and in cases of diabetes insipidus (*n* = 4, 9.1%) (Fig. 2B). However, newly recorded thyrotropic, somatotropic, and gonadotropic deficits at discharge should be interpreted with caution; due to the dynamic nature of these hormones, they may represent pre-existing rather than treatment-induced deficiencies.

By the first follow-up (which took place about 6 weeks postoperatively, see Methods), corticotropic axis deficits comprised 47.1% (*n* = 24/51 of new EDs), while thyrotropic axis deficits decreased to 19.6% (*n* = 10/51, Fig. 2B). Deficits in the somatotropic and gonadotropic axes were observed in 7.8% (*n* = 4/51) and 9.8% (*n* = 5/51) of patients, respectively, and the incidence of DI increased to 15.7% (*n* = 8/51, Fig. 2B). New EDs stratified by additional resection upon ioMRI are illustrated in Fig. 2C.

At the final follow-up (median final follow-up timepoint was 4.1 years, since surgery see Methods), corticotropic dysfunction comprised 34.3% (*n* = 12/35, mainly occurring as the only remaining ED, see upset plot in Fig. 2D), thyrotropic 31.4% (*n* = 11/35), somatotropic 8.6% (*n* = 3/35), and gonadotropic 14.3% (*n* = 5/35) of new EDs while DI was present in 11.4% (*n* = 4/35) of patients with new EDs (Fig. 2B). Figure 2D further depicts the co-occurrence patterns of newly developed EDs within patients.

### Risk factors for new endocrinological deficits

In univariate logistic regression analysis, male sex was associated with a risk of developing a new ED at the last follow-up (OR 0.37, 95% CI 0.16–0.85, *p* = 0.021). None of the other evaluated clinical or surgical variables were significantly associated with new ED at discharge or at final follow-up. Particularly, additional resection after ioMRI did not increase the odds of new EDs (OR at last follow-up 0.88, 95% CI 0.38-2.00, *p* = 0.8) (Supplementary Table 4).

In a multivariate logistic regression model, additional resection after ioMRI was not associated with an increased ratio of new EDs at the last follow-up (OR 1.15, 95% CI 0.43–3.12, *p* = 0.8). Other variables, including sex, age, intended extent of resection, adenoma volume, and residual volume after ioMRI, as well as preexisting endocrine dysfunction, were also not significantly associated with new EDs (Table 3).

**Table 3:** Multivariate penalized logistic regression: Risk of developing a new endocrinological deficit

	New endocrinological deficit								
Characteristic	at discharge			at first FU			at last FU		
	OR	95% CI	*p*-value	OR	95% CI	*p*-value	OR	95% CI	*p*-value
Apoplexy									
no	—	—		—	—		—	—	
yes	2.62	0.69, 9.67	0.2	0.69	0.12, 2.96	0.6	2.17	0.44, 9.19	0.3
Sex									
Female	—	—		—	—		—	—	
Male	0.66	0.28, 1.54	0.3	0.58	0.25, 1.35	0.2	0.50	0.20, 1.22	0.13
Age	1.01	0.98, 1.05	0.5	1.01	0.98, 1.05	0.5	1.04	1.00, 1.08	0.074
Zurich Pituitary Score									
I	—	—		—	—		—	—	
II	1.89	0.31, 20.8	0.5	1.11	0.21, 7.49	> 0.9	0.72	0.13, 5.09	0.7
III	2.55	0.23, 40.8	0.5	0.19	0.01, 2.70	0.2	0.36	0.02, 5.18	0.5
IV	2.06	0.08, 53.9	0.7	0.72	0.03, 14.1	0.8	1.73	0.08, 39.9	0.7
Zurich Pituitary Score ratio	0.97	0.12, 6.89	> 0.9	3.36	0.40, 28.9	0.3	1.65	0.18, 13.8	0.6
Intended extent of resection									
STR	—	—		—	—		—	—	
GTR	2.03	0.65, 7.63	0.2	2.07	0.65, 8.02	0.2	3.83	0.95, 24.6	0.060
Tumor volume	1.03	0.95, 1.11	0.4	1.04	0.97, 1.13	0.3	1.04	0.96, 1.13	0.3
Residual tumor volume ioMRI	1.02	0.89, 1.17	0.7	1.01	0.88, 1.18	0.9	0.99	0.80, 1.14	> 0.9
Additional resection after ioMRI									
no	—	—		—	—		—	—	
yes	2.15	0.93, 5.19	0.073	1.30	0.57, 3.03	0.5	1.13	0.45, 2.87	0.8
ED preop									
no	—	—		—	—		—	—	
yes	0.67	0.24, 1.89	0.4	0.48	0.18, 1.25	0.13	0.48	0.16, 1.42	0.2

Firth’s penalized likelihood logistic regression was used

*ED endocrinological deficit; FU follow-up; STR subtotal resection; GTR gross total resection; ioMR intraoperative MRI; OR odds ratio; CI confidence interval*

To account for baseline selection bias, a sensitivity analysis was conducted within a propensity score-matched cohort subset. Following covariate balancing (Supplementary Fig. 2), the matched sub-cohort was analyzed to evaluate the specific association of additional surgical resection on new endocrinological deficits. Notably, neither in the uni- nor in the multivariate penalized logistic regression models were an additional resection after ioMRI found to be significantly associated with the development of new endocrinological deficits at any examined timepoint (Supplementary Tables 5 and 6). These matched findings strongly confirm the robustness of our primary whole-cohort analysis.

## Discussion

The central objective of the present study was to determine whether the pursuit of maximal resection using ioMRI increases the risk of these deficits. Our data demonstrate that the rate of new permanent EDs of at least one axis at final follow-up was 18%, a figure that remains well within the reported literature range of up to 35% [5, 15, 16, 17, 18, 19, 20, 21, 22, 23, 24, 25, 26, 27, 28, 29, 30, 31, 32, 33] (Supplementary Table 7). No correlation was found between patients who showed recovery of pituitary function and additional resection with ioMRI. Notably, although ioMRI-guided additional resection enabled the conversion of subtotal resection to GTR in up to 74% of cases, this extension of the surgical procedure did not correlate with an increased risk of permanent hormonal impairment.

These findings align with recent evidence from Zhang et al. [20] who reported no increased incidence of hypopituitarism following ioMRI-guided additional resection in a cohort of 133 NFPA cases. Similarly, both Hlavac et al. [4] and Berkmann et al. [24] observed that the intraoperative use of MRI did not adversely affect hormonal axis recovery or increase the rate of new endocrinological deficits compared to standard techniques. However, it is critical to note that the existing literature on this topic frequently relies on older microsurgical series [24], or highly heterogeneous cohorts that mix invasive adenomas (Knosp grades III–IV) with various functional subtypes resected via a combination of microsurgical and endoscopic approaches [4]. Consequently, our study significantly adds to the current body of evidence by evaluating a large, contemporary, and highly homogeneous cohort treated strictly with modern endoscopic techniques for NFPAs.

Assessing endocrinological outcomes following TSS for NFPAs is essential for refining surgical techniques and providing accurate preoperative counseling. However, a primary challenge in the current literature is the profound inconsistency in how endocrinological outcomes are reported. Reported rates of new postoperative EDs fluctuate dramatically, ranging from 1.4% to 35% (Supplementary Table 3), while the recovery of previously deficient axes is cited between 11% and 98% [34], with a recent study indicating that greater extent of tumor resection is associated with improved recovery of previously deficient hormonal axes [35]. This high degree of variability is largely attributable to heterogeneous study cohorts and retrospective assessments of endocrine function which are susceptible to reporting bias. Furthermore, there is a distinct lack of standardized definitions for hormonal failure; while some centers define ED based strictly on biochemical laboratory thresholds [15–27] others utilize a more pragmatic, clinically-driven definition based on the initiation of new hormone replacement therapy [28–30, 36]. The number of hormonal axes evaluated, co-occurrences of partial recoveries and newly impaired hormone axes and the duration of follow-up—which varies from not reported at all to 60 months—further obscure direct comparisons between surgical cohorts (Supplementary Table 7).

The lack of standardized reporting time points remains a confounding factor, as hormonal recovery is a dynamic process where transient early deficits may resolve, or delayed-onset dysfunctions may emerge months postoperatively. In this study, we addressed the potential for bias related to perioperative corticosteroid prophylaxis—which may temporarily mask true ACTH-axis function—by focusing on the long-term assessment of hormone axes. This approach provides a functionally relevant benchmark for surgical morbidity. Our results suggest a critical clinical implication: ioMRI facilitates a safer trajectory toward achieving GTR - which is well-established to reduce the risk of tumor recurrence [3, 5, 6, 37] - without compromising endocrine function. Crucially, this safety profile holds true across the entire postoperative course, showing no increased risk for either short-term transient disturbances or long-term permanent hormonal deficiencies. However, the balance between aggressive resection and the preservation of the hormonal axes must remain a tailored surgical priority.

### Limitations

Our study has several limitations that warrant consideration. First, its retrospective, non-randomized design at a single center introduces inherent risks of selection bias, particularly regarding the individualized intraoperative decision to pursue an additional resection following ioMRI findings. Although we utilized a propensity score-matched sensitivity analysis to control for key confounding variables, unmeasured clinical or anatomical confounders may still influence these findings. Second, while our cohort represents a highly homogeneous and comprehensively evaluated population of NFPAs, the total number of patients who experienced new endocrinological deficits was relatively small. This limited sample size restricts our overall statistical power to detect subtle, rare, or axis-specific hormonal changes. Finally, our findings reflect the surgical philosophy and experience of a specialized skull base team at a high-volume academic tertiary care center, which may limit the direct generalizability of our results to other centers with e.g. alternative surgical frameworks.

## Conclusion

In summary, the use of ioMRI during TSS for NFPA facilitates the conversion of a substantial percentage of cases to GTR without an associated increase in the risk of permanent new endocrinological deficits. While the safety of ioMRI-guided additional resection is demonstrated in our cohort, the interpretation of endocrine outcomes across the broader literature remains fundamentally challenged by the lack of standardized reporting. Significant discrepancies in how new deficits and hormonal recovery are defined limit the comparability of multi-institutional data. Establishing standardized assessment protocols with fixed postoperative time points and unified diagnostic criteria as well as prospective data collection is essential to improve the consistency of clinical reporting. Such advancements would allow for more robust benchmarks across centers and, crucially, provide patients with more reliable expectations regarding procedural risk of hormone replacement therapy. 

## Electronic Supplementary Material

Below is the link to the electronic supplementary material.

Supplementary Material 1 (DOCX 82.9 KB )

## Data Availability

Raw data and R scripts for the figures and analyses are available from the corresponding author upon reasonable request.
