# Supplementary material for: Risk of new endocrinological deficits after intraoperative MRI-guided additional resection in endoscopic non-functioning pituitary adenoma surgery
Source: Pituitary. 2026 Jul 11;29(4):123. doi: 10.1007/s11102-026-01727-0 (PMC13356093; doi:10.1007/s11102-026-01727-0)
Supplement: Supplementary file 1 — Supplementary Material 1 (DOCX 82.9 KB ) [file 11102_2026_1727_MOESM1_ESM.docx]

**SUPPLEMENTARY FIGURES**


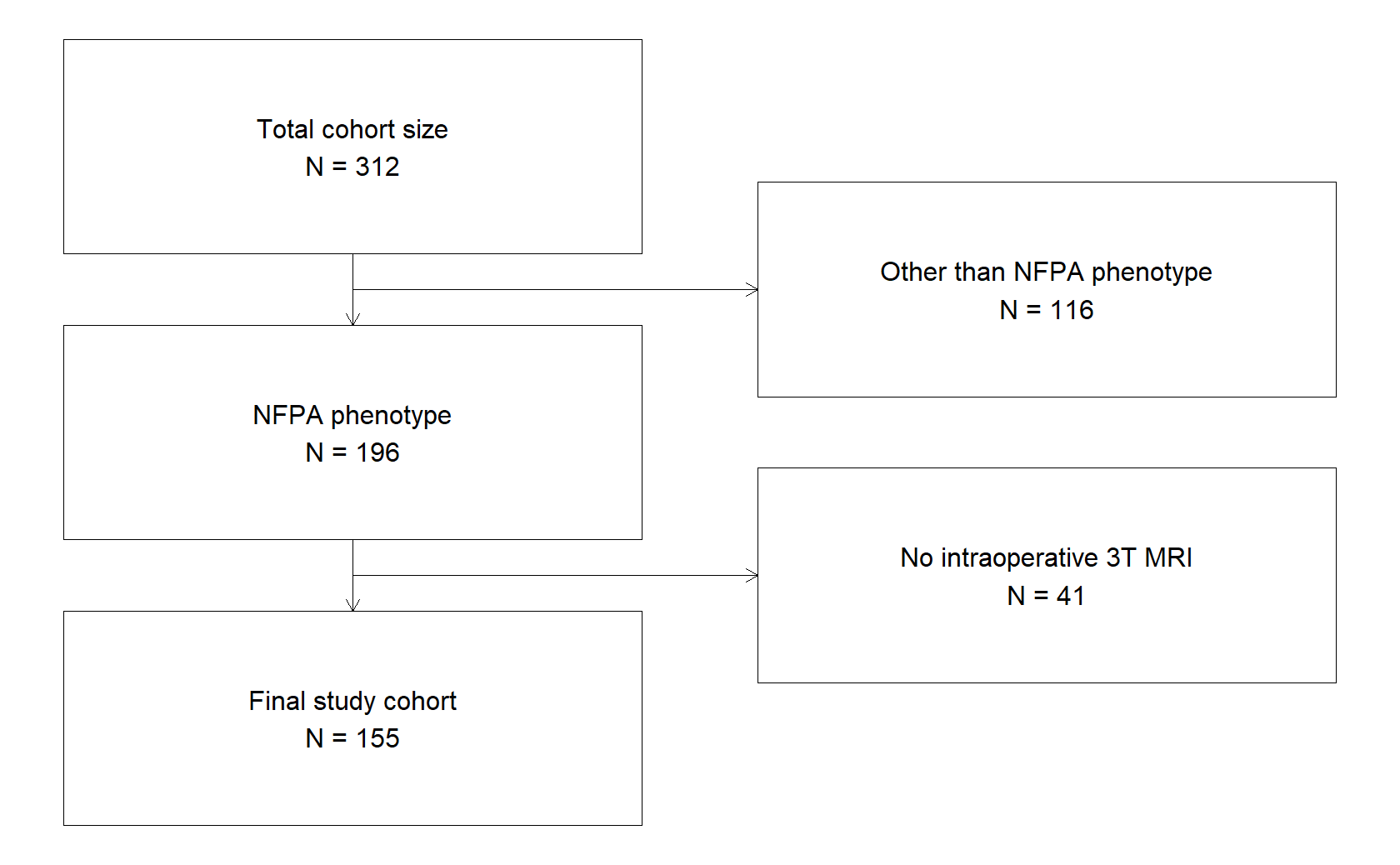


**Supplementary Figure 1: Flow-chart Inclusion criteria**


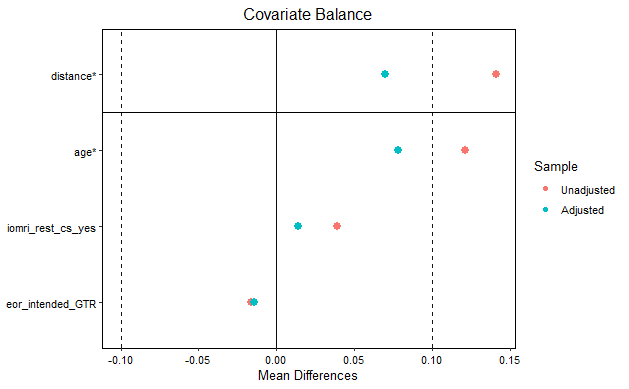


**Supplementary Figure 3: Covariate balance plot before and after propensity score matching.**

The plot displays the standardized mean differences for the baseline covariates between the additional resection and no additional resection groups before matching (Unadjusted) and after 1:1 nearest-neighbor matching (Adjusted). A standardized mean difference close to 0 indicates excellent balance between the cohorts.

*age Age; iomri_rest_cs_yes Residual tumor in the cavernous sinus; eor_intended_GTR Preoperatively intended gross total resection.*

**
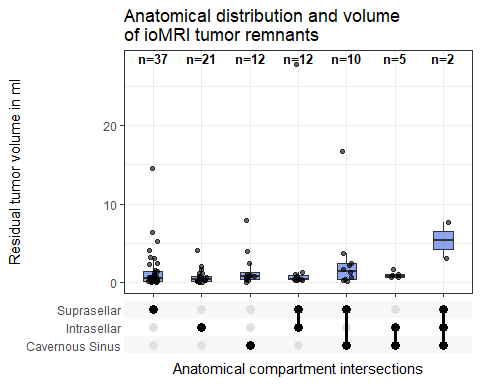
**

**Supplementary Figure 3: Intersections of anatomical tumor remnant compartments and corresponding volumes on intraoperative MRI.** The intersection matrix (bottom panel) illustrates the overlapping distribution of residual tumor across the three principal compartments: intrasellar, suprasellar and cavernous sinus. The overlaid box and jitter plots demonstrate the distribution of the corresponding residual tumor volumes within each distinct intersection profile.

*ioMRI intraoperative MRI*

**SUPPLEMENTARY TABLES**

| **Characteristic** | **N missing (%)*^1^*** |
| --- | --- |
| Sex | 0 (0%) |
| Age | 0 (0%) |
| First transsphenoidal surgery | 9 (5.8%) |
| Apoplexy | 11 (7.1%) |
| Phenotype | 0 (0%) |
| Zurich Pituitary Score | 1 (0.6%) |
| Intended extent of resection | 1 (0.6%) |
| Tumor volume | 3 (1.9%) |
| Additional resection after ioMRI | 0 (0%) |
| Endocrinological deficit preop | 0 (0%) |
| Zurich Pituitary Score ratio | 2 (1.3%) |
| Residual tumor volume ioMRI | 3 (1.9%) |
| *^1^* n (%) | |

**Supplementary Table 1: Missing data**

Absolute number and percentages (%) of missing data per variable.

|  | **Discharge** | | | **First Follow-up** | | | **Last Follow-up** | | |
| --- | --- | --- | --- | --- | --- | --- | --- | --- | --- |
|  | **Additional resection** | |  | **Additional resection** | |  | **Additional resection** | |  |
|  | no N = 53*^1^* | yes N = 50*^1^* | p-value*^2^* | no N = 54*^1^* | yes N = 50*^1^* | p-value*^3^* | no N = 54*^1^* | yes N = 51*^1^* | p-value*^3^* |
| **Any END remission** | 5 (9.4%) | 5 (10%) | >0.9 | 14 (26%) | 18 (36%) | 0.3 | 20 (37%) | 27 (53%) | 0.10 |

**Supplementary Table 2: Endocrinological remissions stratified by additional resection after ioMRI**

P-values were calculated using the Fisher’s exact or Pearson’s Chi-squared test.

*ioMRI intraoperative MRI*

|  | **Discharge** | | | **First Follow-up** | | | **Last Follow-up** | | |
| --- | --- | --- | --- | --- | --- | --- | --- | --- | --- |
|  | **Additional resection** | |  | **Additional resection** | |  | **Additional resection** | |  |
|  | no N = 84*^1^* | yes  N = 71*^1^* | p-value*^2^* | no N = 84*^1^* | yes N = 71*^1^* | p-value*^2^* | no N = 84*^1^* | yes N = 71*^1^* | p-value*^2^* |
| **Any END worsening** | 15 (18%) | 20 (29%) | 0.11 | 21 (25%) | 17 (24%) | >0.9 | 16 (19%) | 12 (17%) | 0.8 |
| **unknown** | 0 | 1 |  | 0 | 1 |  | 0 | 1 |  |

**Supplementary Table 3: New endocrinological deficits stratified by additional resection after ioMRI**

P-values were calculated using the Pearson’s Chi-squared test.

*ioMRI intraoperative MRI*

|  | **New endocrinological deficit** | | | | | | | | |
| --- | --- | --- | --- | --- | --- | --- | --- | --- | --- |
| **Characteristic** | **at discharge** | | | **at first FU** | | | **at last FU** | | |
|  | **OR** | **95% CI** | **p-value** | **OR** | **95% CI** | **p-value** | **OR** | **95% CI** | **p-value** |
| **Apoplexy** |  |  |  |  |  |  |  |  |  |
| no | — | — |  | — | — |  | — | — |  |
| yes | 1.70 | 0.50, 5.12 | 0.4 | 0.45 | 0.07, 1.72 | 0.3 | 1.11 | 0.24, 3.81 | 0.9 |
| **Sex** |  |  |  |  |  |  |  |  |  |
| Female | — | — |  | — | — |  | — | — |  |
| Male | 0.78 | 0.36, 1.67 | 0.5 | 0.53 | 0.25, 1.11 | 0.093 | 0.37 | 0.16, 0.85 | **0.021** |
| **Age** | 1.01 | 0.98, 1.04 | 0.6 | 1.00 | 0.98, 1.03 | 0.8 | 1.02 | 0.99, 1.05 | 0.3 |
| **Zurich Pituitary Score** |  |  |  |  |  |  |  |  |  |
| I | — | — |  | — | — |  | — | — |  |
| II | 3.55 | 0.64, 66.5 | 0.2 | 1.31 | 0.37, 6.20 | 0.7 | 0.81 | 0.22, 3.89 | 0.8 |
| III | 4.71 | 0.78, 91.1 | 0.2 | 0.61 | 0.13, 3.27 | 0.5 | 0.38 | 0.07, 2.19 | 0.3 |
| IV | 3.43 | 0.28, 82.1 | 0.3 | 1.67 | 0.24, 11.9 | 0.6 | 1.67 | 0.24, 11.9 | 0.6 |
| **Zurich Pituitary Score ratio** | 1.43 | 0.46, 4.14 | 0.5 | 1.06 | 0.34, 3.04 | >0.9 | 0.76 | 0.19, 2.52 | 0.7 |
| **Intended extent of resection** |  |  |  |  |  |  |  |  |  |
| STR | — | — |  | — | — |  | — | — |  |
| GTR | 1.50 | 0.60, 4.34 | 0.4 | 1.36 | 0.56, 3.66 | 0.5 | 1.89 | 0.67, 6.83 | 0.3 |
| **Tumor volume** | 1.02 | 0.97, 1.08 | 0.4 | 1.03 | 0.97, 1.08 | 0.3 | 1.00 | 0.93, 1.06 | >0.9 |
| **Residual tumor volume ioMRI** | 1.03 | 0.90, 1.16 | 0.6 | 1.03 | 0.90, 1.15 | 0.6 | 0.96 | 0.74, 1.10 | 0.6 |
| **Additional resection after ioMRI** |  |  |  |  |  |  |  |  |  |
| no | — | — |  | — | — |  | — | — |  |
| yes | 1.84 | 0.86, 4.00 | 0.12 | 0.96 | 0.46, 2.01 | >0.9 | 0.88 | 0.38, 2.00 | 0.8 |
| **ED preop** |  |  |  |  |  |  |  |  |  |
| no | — | — |  | — | — |  | — | — |  |
| yes | 1.20 | 0.53, 2.95 | 0.7 | 0.60 | 0.28, 1.32 | 0.2 | 0.67 | 0.28, 1.63 | 0.4 |

**Supplementary Table 4: Univariate logistic regression: Risk of developing a new endocrinological deficit**

*ED endocrinological deficit; FU follow-up; STR subtotal resection; GTR gross total resection; ioMR intraoperative MRI; OR odds ratio; CI confidence interval*

|  | **New endocrinological deficit** | | | | | | | | |
| --- | --- | --- | --- | --- | --- | --- | --- | --- | --- |
| **Characteristic** | **at discharge** | | | **at first FU** | | | **at last FU** | | |
|  | **OR** | **95% CI** | **p-value** | **OR** | **95% CI** | **p-value** | **OR** | **95% CI** | **p-value** |
| **Apoplexy** |  |  |  |  |  |  |  |  |  |
| no | — | — |  | — | — |  | — | — |  |
| yes | 1.69 | 0.49, 5.19 | 0.4 | 0.47 | 0.07, 1.84 | 0.3 | 1.15 | 0.25, 4.03 | 0.8 |
| **Sex** |  |  |  |  |  |  |  |  |  |
| Female | — | — |  | — | — |  | — | — |  |
| Male | 0.90 | 0.41, 2.02 | 0.8 | 0.59 | 0.27, 1.31 | 0.2 | 0.49 | 0.20, 1.18 | 0.11 |
| **Age** | 1.00 | 0.97, 1.03 | 0.9 | 1.00 | 0.97, 1.03 | 0.8 | 1.01 | 0.98, 1.05 | 0.5 |
| **Zurich Pituitary Score** |  |  |  |  |  |  |  |  |  |
| I | — | — |  | — | — |  | — | — |  |
| II | 3.17 | 0.56, 60.1 | 0.3 | 1.83 | 0.43, 12.6 | 0.5 | 1.16 | 0.27, 8.07 | 0.9 |
| III | 3.70 | 0.59, 72.4 | 0.2 | 0.70 | 0.13, 5.48 | 0.7 | 0.55 | 0.09, 4.38 | 0.5 |
| IV | 1.43 | 0.05, 40.4 | 0.8 | 1.50 | 0.15, 15.6 | 0.7 | 1.50 | 0.15, 15.6 | 0.7 |
| **Zurich Pituitary Score ratio** | 1.16 | 0.35, 3.52 | 0.8 | 1.07 | 0.32, 3.24 | >0.9 | 0.89 | 0.22, 3.05 | 0.9 |
| **Intended extent of resection** |  |  |  |  |  |  |  |  |  |
| STR | — | — |  | — | — |  | — | — |  |
| GTR | 1.73 | 0.65, 5.52 | 0.3 | 1.39 | 0.54, 4.06 | 0.5 | 2.39 | 0.75, 10.6 | 0.2 |
| **Tumor volume** | 1.02 | 0.96, 1.07 | 0.6 | 1.03 | 0.98, 1.09 | 0.3 | 1.00 | 0.92, 1.05 | 0.9 |
| **Residual tumor volume ioMRI** | 1.03 | 0.90, 1.15 | 0.6 | 1.03 | 0.91, 1.15 | 0.6 | 0.96 | 0.73, 1.10 | 0.6 |
| **Additional resection after ioMRI** |  |  |  |  |  |  |  |  |  |
| no | — | — |  | — | — |  | — | — |  |
| yes | 1.67 | 0.75, 3.78 | 0.2 | 0.94 | 0.43, 2.06 | 0.9 | 0.76 | 0.31, 1.81 | 0.5 |
| **ED preop** |  |  |  |  |  |  |  |  |  |
| no | — | — |  | — | — |  | — | — |  |
| yes | 1.06 | 0.44, 2.77 | 0.9 | 0.62 | 0.27, 1.48 | 0.3 | 0.69 | 0.28, 1.85 | 0.4 |

**Supplementary Table 5: Univariate logistic regression of propensity score matched cohort: Risk of developing a new endocrinological deficit**

*ED endocrinological deficit; FU follow-up; STR subtotal resection; GTR gross total resection; ioMR intraoperative MRI; OR odds ratio; CI confidence interval*

|  | **New endocrinological deficit** | | | | | | | | |
| --- | --- | --- | --- | --- | --- | --- | --- | --- | --- |
| **Characteristic** | **at discharge** | | | **at first FU** | | | **at last FU** | | |
|  | **OR** | **95% CI** | **p-value** | **OR** | **95% CI** | **p-value** | **OR** | **95% CI** | **p-value** |
| **Apoplexy** |  |  |  |  |  |  |  |  |  |
| no | — | — |  | — | — |  | — | — |  |
| yes | 2.53 | 0.66, 9.40 | 0.2 | 0.66 | 0.11, 2.86 | 0.6 | 2.12 | 0.43, 9.03 | 0.3 |
| **Sex** |  |  |  |  |  |  |  |  |  |
| Female | — | — |  | — | — |  | — | — |  |
| Male | 0.70 | 0.29, 1.65 | 0.4 | 0.64 | 0.26, 1.53 | 0.3 | 0.60 | 0.24, 1.52 | 0.3 |
| **Age** | 1.01 | 0.97, 1.05 | 0.6 | 1.00 | 0.97, 1.04 | 0.8 | 1.03 | 0.99, 1.07 | 0.12 |
| **Zurich Pituitary Score** |  |  |  |  |  |  |  |  |  |
| I | — | — |  | — | — |  | — | — |  |
| II | 1.91 | 0.31, 21.4 | 0.5 | 1.85 | 0.28, 21.3 | 0.5 | 1.20 | 0.17, 14.1 | 0.9 |
| III | 2.50 | 0.22, 40.3 | 0.5 | 0.34 | 0.02, 6.73 | 0.5 | 0.57 | 0.03, 11.5 | 0.7 |
| IV | 2.02 | 0.08, 53.6 | 0.7 | 1.13 | 0.05, 30.5 | >0.9 | 2.73 | 0.11, 82.1 | 0.5 |
| **Zurich Pituitary Score ratio** | 0.90 | 0.11, 6.41 | >0.9 | 3.22 | 0.37, 28.2 | 0.3 | 1.60 | 0.17, 13.8 | 0.7 |
| **Intended extent of resection** |  |  |  |  |  |  |  |  |  |
| STR | — | — |  | — | — |  | — | — |  |
| GTR | 1.94 | 0.61, 7.32 | 0.3 | 1.86 | 0.57, 7.25 | 0.3 | 3.63 | 0.90, 23.1 | 0.072 |
| **Tumor volume** | 1.03 | 0.95, 1.11 | 0.5 | 1.04 | 0.97, 1.12 | 0.3 | 1.04 | 0.95, 1.12 | 0.3 |
| **Residual tumor volume ioMRI** | 1.02 | 0.89, 1.17 | 0.7 | 1.02 | 0.89, 1.18 | 0.8 | 1.00 | 0.81, 1.15 | >0.9 |
| **Additional resection after ioMRI** |  |  |  |  |  |  |  |  |  |
| no | — | — |  | — | — |  | — | — |  |
| yes | 1.82 | 0.78, 4.43 | 0.2 | 1.22 | 0.51, 2.95 | 0.7 | 0.99 | 0.39, 2.57 | >0.9 |
| **ED preop** |  |  |  |  |  |  |  |  |  |
| no | — | — |  | — | — |  | — | — |  |
| yes | 0.66 | 0.24, 1.85 | 0.4 | 0.52 | 0.19, 1.42 | 0.2 | 0.47 | 0.16, 1.41 | 0.2 |

**Supplementary Table 6: Multivariate penalized logistic regression of propensity score matched cohort: Risk of developing a new endocrinological deficit**

Firth’s penalized likelihood logistic regression was used.

*ED endocrinological deficit; FU follow-up; STR subtotal resection; GTR gross total resection; ioMR intraoperative MRI; OR odds ratio; CI confidence interval*

| **Author (Year)** | **No. of Patients** | **NFPA/FPA** | **New ED postop** | **ED definition** | **Recovery Rate** | **FU-duration** |
| --- | --- | --- | --- | --- | --- | --- |
| Guerra et al. (2026) | 524 | Both | 185/524 (35%) | Additional medication postoperatively | 8.2% GH; 3.8% ACTH; 7% PRL | 6 weeks, 3 months; mean/median not specified |
| Bander et al. (2025) | 372 | NFPA | 30.8% | Post-operative hormone replacement | 39.5% | ≥3 months, mean not specified |
| Araujo-Castro et al. (2022) | 146 | Both | 13.4% | Lab values or new hormone replacement | 56.9% | Variable, 4-12 months |
| Quah et al. (2022) | 137 | NFPA | 24.7–31.8% | Lab values + symptoms | Not reported | 10.5–13 months |
| Choo et al. (2022) | 181 | NFPA | Not reported | Clinic + Lab values | 77.5% | Median 58 months |
| Castle-Kirszbaum et al. (2022) | 304 | Both | 8% | Endocrinologist confirmed deficit according to lab values | 15.9% | Not explicitly defined |
| Gerges et al. (2021) | 190 | NFPA | Not reported | Lab values | Not reported | ≥5 years |
| Alexopoulou et al. (2021) | 246 | NFPA | 25/175 (14%) in patients with preoperative dysfunction; 10/44 (23%) in patients with normal axes; overall: 35/219 (16%) | Clinic + Lab values | 50% | 1 year |
| Vivancos Sánchez et al. (2021) | 117 | NFPA | 19.8% | Lab values | 13% normalization; 16.7% improvement | 12 months |
| Galloway et al. (2021) | 145 | Both | 18% | New hormonal deficiency requiring HRT | 11% discontinued HRT | Median 52 months |
| Hwang et al. (2020) | 209 | NFPA | 17.2% | Lab values | 29.7% | 12 months |
| Yi et al. (2019) | 67 | Both | 1 patient worsened | Clinic + Lab values | 62.7% | ≥3 months, mean not specified |
| Zhang et al. (2019) | 164 | NFPA | Not specified | Lab values | Varied by axis: TSH 100%, IGF1 77.8% | 21.1 ± 3.3 months |
| Harary et al. (2019) | 160 | NFPA | Not directly specified | Lab values | 55% recovery of at least one axis | Median 24.4 months |
| Hlaváč et al. (2019) | 111 | Both | 22.1% new hypopituitarism, 10.5% new diabetes insipidus | Endocrinologist-confirmed deficit based on lab values and clinical data | Not reported | 3–12 months |
| Kim et al. (2018) | 331 | NFPA | 32.9% | New post-operative hormone replacement | 15.4% | 3 months + long-term not detailed |
| Jahangiri et al. (2016) | 305 | NFPA | 13.7% | Lab values | 73/111 (66%) recovery of at least one axis | 6 weeks – 6 months |
| Marenco et al. (2015) | 25 | NFPA (elderly patients) | Not specified | Lab values | 22.2% | Mean 6.7 days inpatient, FU unclear |
| Gondim et al. (2015) | 55 | NFPA (elderly patients) | 12.7% | Lab values | Not reported | Mean 50 months |
| Mamelak et al. (2013) | 300 | Both | 1.4% | Lab values | Not reported | Mean 37 ± 22 months |
| Berkmann et al. (2012) | 60 | NFPA | 29% | Lab values | 59% | Mean 3.2 years |
| Messerer et al. (2011) | 164 | NFPA | 22% | Endocrinological follow-up with lab values | 56% improved in endoscopic group | 12 months |
| Gondim et al. (2010) | 228 | Both | Not reported | Lab values assessed at 3 months | 83% recovery in NFPA | ≥3 months |
| Fatemi et al. (2008) | 444 | Both | 5.5% (all adenomas); 7.4% (NFPA only) | Lab values or hormone replacement at 3 months | Any recovery in 49% (all adenomas); 48% (NFPA only) | Median follow-up 16 months |
| Nomikos et al. (2004) | 721 | NFPA | 1.4% | Lab values | 30.1% | 1 year |

**Supplementary Table 7: Reported endocrinological deficits after TSS in the literature**

*FU follow-up; (N)FPA (non-)functioning pituitary adenoma; TSS transsphenoidal surgery*

**SUPPLEMENTARY METHODS**

## Standard Endoscopic Transsphenoidal Surgical Technique

**Nasal phase:** A right-sided mononostril endoscopic approach was systematically performed. Following the identification of anatomical landmarks, the right middle and superior turbinates were lateralized, and the sphenoid ostium was identified. At the level of the ostium, a septal mucosal incision was performed in case there was need to harvest a rescue flap. A posterior septostomy and wide sphenoidotomy were then performed, followed by lateralization of the posterior septum, identification of the contralateral sphenoid ostium, and communication of the two ostia to create a unified surgical corridor.

**Sphenoid phase:** Following exposure of the sphenoid rostrum, the midline, intersphenoid septations, and any anatomical variants were identified. The sphenoid sinus mucosa was removed and obstructing bony septations were carefully removed to maximize visual exposure.

**Sellar phase:** Adequate exposure of all critical landmarks was verified, including the clival recess, the bilateral opticocarotid recesses, the internal carotid artery protuberances, the planum sphenoidale, and the tuberculum sellae; optical neuronavigation was utilized adjunctively when needed. The sellar floor opening was executed using a chisel, followed by a sharp dural incision. Adenoma resection was performed using a piecemeal technique. In macroadenomas with significant suprasellar extension, the inferior and lateral compartments were resected first to facilitate the gravity-induced descent of the suprasellar tumor component. Prior to or following ioMRI, the resection boundaries were inspected using angled endoscopes or via high-magnification close-up inspection under continuous irrigation ("diving technique").

**IoMRI:** To optimize radiologic contrast on the ioMRI imaging sequences, the surgical cavity was temporarily filled with a gelatin-thrombin matrix (FloSeal, Baxter Healthcare). A sponge packing was placed in the nasal cavity. Standardized institutional ioMRI safety checklists and patient draping protocols were completed prior to transferring and scanning the patient.

**Closure phase:** Following the confirmation of meticulous hemostasis and clearance of surgical debris, the sellar defect was reconstructed. In the absence of a CSF leak, a two-layer absorbable gelatin sponge was applied over the sellar opening and reinforced with fibrin glue. In the event of an intraoperative CSF leak, reconstruction was upgraded to a multilayer closure consisting of an autologous fat graft and a fasciae lata graft (harvested from the left thigh) utilized in an inlay/outlay fashion. Finally, standard nasal packing was placed.
